# Wnt Signaling in Thyroid Homeostasis and Carcinogenesis

**DOI:** 10.3390/genes9040204

**Published:** 2018-04-10

**Authors:** Kim A. Ely, Lindsay A. Bischoff, Vivian L. Weiss

**Affiliations:** 1 Department of Pathology, Microbiology, and Immunology, Vanderbilt University Medical Center, Nashville, TN 37232, USA; kim.ely@vanderbilt.edu; 2 Department of Medicine, Vanderbilt University Medical Center, Nashville, TN 37232, USA; lindsay.bischoff@vanderbilt.edu

**Keywords:** thyroid cancer, Wnt signaling, cancer stem cells

## Abstract

The Wnt pathway is essential for stem cell maintenance, but little is known about its role in thyroid hormone signaling and thyroid stem cell survival and maintenance. In addition, the role of Wnt signaling in thyroid cancer progenitor cells is also unclear. Here, we present emerging evidence for the role of Wnt signaling in somatic thyroid stem cell and thyroid cancer stem cell function. An improved understanding of the role of Wnt signaling in thyroid physiology and carcinogenesis is essential for improving both thyroid disease diagnostics and therapeutics.

## 1. Introduction

Somatic (adult) stem cells are necessary for homeostasis across a variety of tissues. Among the best-characterized examples are the gastrointestinal tract, where there is rapid turnover of the epithelium [1,2], and hematopoietic tissues, which have a strong capacity to regenerate that is dependent on the presence of long-term somatic stem cells [3]. In both cases, Wnt signaling is needed for stem cell survival and for the upkeep of their tissue regenerative capacity [1,4]. While normal thyroid tissue has a low turnover rate, during times of injury, it has an immense capability for cellular renewal. Given the critical nature of Wnt signaling in the maintenance of stem cells in other tissues [5,6], it is not surprising that Wnt signaling has been implicated in thyroid development, homeostasis, and carcinogenesis [7,8].

Thyroid disease affects 20 million Americans, with approximately one in 20–30 people in the United States diagnosed with hypothyroidism, or insufficient thyroid hormone [9]. Artificial thyroid hormone replacement with levothyroxine is the standard of care for hypothyroidism. However, despite this treatment, individuals with such thyroid dysfunction suffer from imbalances in cell metabolism, which can affect many important tissues throughout the body, including skin, bones, brain, and intestine. Thus, the management of exogenous thyroid hormone is of critical importance and remains a challenge.

Thyroid hormone (TH) plays a role in many cellular functions, including proliferation, growth, differentiation, metabolism, regeneration, and homeostasis. It is produced by the thyroid gland and then travels through the bloodstream to regulate gene transcription at diverse sites. While the hypothalamic/pituitary/thyroid axis controls thyroid hormone synthesis, TH is so critical for cellular function that it is also tightly regulated at the level of the individual tissues. Thyroid hormone enters cells by membrane transport and is modified by deiodinases on its path from the membrane to the nucleus. By catalyzing the release of iodine from thyroid hormones, deiodinases allow for tissue-specific regulation of cellular levels of active thyroid hormone. Each tissue tightly controls the expression of various deiodinase isoforms to allow for precise regulation of active thyroid hormone concentrations. Once thyroid hormone reaches the nucleus, it binds to its nuclear receptors, which regulate gene transcription. Thyroid hormone receptors (TRs) provide another level of cellular control of TH actions. Recent evidence suggests that Wnt/β-catenin signaling (henceforth simply “Wnt signaling”) is involved in regulating the expression of TRs and deiodinases in the target tissues to allow for TH-driven transcriptional programs.

While somatic stem cells have been well-characterized in a variety of tissues, their function in thyroid regeneration and thyroid cancer is just beginning to be described. In addition, our understanding of the role of Wnt signaling in both normal and neoplastic thyroid epithelial proliferation is evolving. Herein, we review the development and physiology of the thyroid gland and the emerging evidence for the role of Wnt signaling in thyroid development, maintenance of adult thyroid stem cells, and thyroid cancer. Finally, we discuss the future directions for defining the role of the Wnt pathway in thyroid stem cell research and the important questions that remain to be answered.

## 2. Thyroid Physiology and the Molecular Mechanisms of Thyroid Hormone Function

The thyroid gland is essential for organism homeostasis due to its role in the production of TH. TH is controlled by the hypothalamic/pituitary/thyroid axis, leading to downstream stimulation of its production and regulation by a feedback mechanism. However, TH is also tightly monitored at the level of the individual tissues. There is evidence that Wnt signaling plays a role in thyroid hormone function and regulation.

### 2.1. Thyroid Hormone Regulation and Signaling

TH has long been known to regulate metabolism. Patients with TH dysfunction often display the symptoms of metabolic dysregulation, including fatigue and weight changes. TH synthesis is controlled by the hypothalamic/pituitary/thyroid axis. Thyrotropin-releasing hormone (TRH) is synthesized in the hypothalamus and migrates to the anterior pituitary through the portal capillary plexus [10]. TRH receptors are present within the membranes of anterior pituitary cells, and their stimulation leads to release of thyroid-stimulating hormone (TSH). TSH is responsible for regulating TH secretion by the thyroid gland. It binds to the membrane receptor (TSHR) in the thyroid gland and stimulates production of TH, the Na^+^/I^−^ symporter (NIS), thyroglobulin (Tg), and thyroid peroxidase (TPO). NIS transports iodide into the cell, which is oxidized by TPO and incorporated into Tg. Monoiodinated and diiodinated Tg residues are enzymatically coupled to form thyroxine (T4) and triiodothyronine (T3) [10]. T4 and T3 are then emitted into the blood stream. T4 is the major form and is 40-fold higher in the serum than the active form, T3. T4 is catalyzed to its active form, T3, in peripheral cells. Most TH travels through the circulation bound to thyroxine-binding globulin (TBG), albumin, or thyroid-binding prealbumin [10]. Once TH enters the cell, T3 binds to nuclear thyroid hormone receptors, which heterodimerize and bind to thyroid hormone response elements (TREs) in the promoter regions of genes, thereby enhancing transcription of target genes [11].

While plasma concentrations of TH are relatively stable, tissues can coordinate different TH levels due to their regulation of deiodinases and thyroid hormone receptors. The iodothyronine deiodinase family of selenoproteins are enzymes that include D1, D2, and D3. These deiodinases are present in specific tissues in order to regulate thyroid hormone activation and inactivation. The differential expression of deiodinases allows for tight control of T3 and its prohormone, T4 [12]. T4 has a longer half-life and is converted to the active form, T3, within cells through deiodinases. D1 is the most abundant deiodinase and is found predominantly in liver and kidney, and to a lesser extent in skeletal muscle, heart, and thyroid tissue (Table 1). Because its major goal is to release active thyroid hormone, T3, into the circulation, it converts T4 from the thyroid into T3 by deiodination of the T4 outer ring [13]. In hyperthyroidism, D1 function is increased, resulting in supraphysiologic T3 levels in the circulation. D2 is responsible for the intracellular conversion of T4 to the active form T3, by 5′-deoiodination or removal of iodine. D2 is negatively regulated by thyroid hormone in order to increase T4 to T3 conversion within the target tissues during times of low thyroid hormone [12,14]. D2 is predominantly expressed in the central nervous system (CNS) to regulate active thyroid hormone levels in the brain (Table 1). D3 is responsible for inactivation of thyroid hormones to maintain homeostasis. D3 deiodinates the inner ring of the T4 molecule, resulting in 3,3′,5′-triiodothyronine, or reverse T3. D3 also deiodinates T3 to 3,3′-diiodothyronine, or T2. D3, predominantly expressed in the placenta and brain, is elevated in hyperthyroidism, thereby serving a protective function from fetal hyperthyroidism and maintaining CNS euthyroidism (Table 1). Similarly, D3 function is decreased in hypothyroidism to maintain active homeostasis.

**Figure 1:**
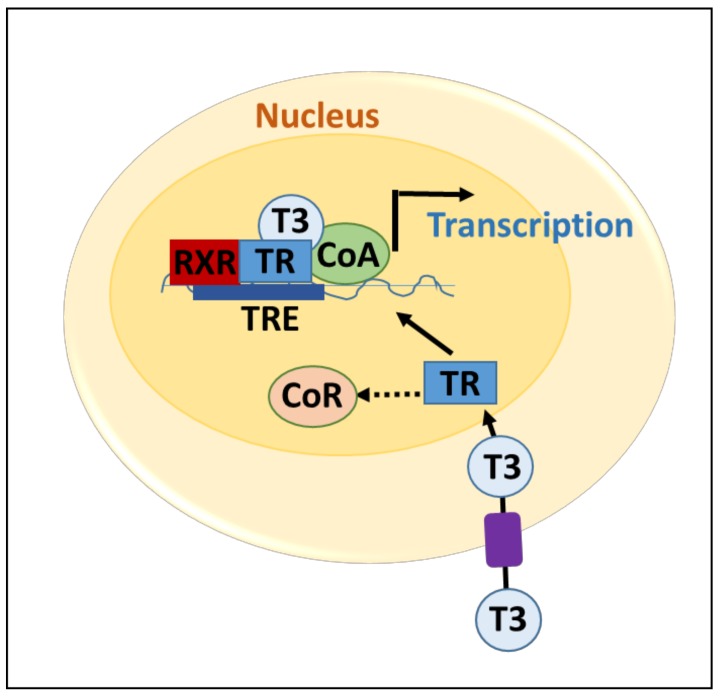
Thyroid hormone receptor-mediated transcription. T3 enters the target cell through membrane transport. T3 enters the nucleus and binds to the thyroid hormone receptor (TR). TR then releases the co-repressor (CoR), dimerizes with the retinoid X receptor (RXR), and recruits the co-activator (CoA) complex. This complex binds the T3 response element (TRE) to activate transcription of target genes.

TH action can also be controlled at the cellular level by the expression of various thyroid hormone receptors. TR mRNA can be variably spliced to form TRα (TRα1, TRα2, and TRα3) and TRβ (TRβ1 and TRβ2) [15]. These receptors can heterodimerize and bind to regulatory regions of DNA with the retinoid X receptor (RXR) (Figure 1) [11]. They can also form less transcriptionally active units as homodimers or monomers when they bind DNA [15]. Tissue-specific expression of the various isoforms helps to regulate the level of TH signaling as well as the proliferative or differentiating function of TH action.

Recently, dynamic regulation of TH at the tissue level has been demonstrated to be critical not only in normal development, but also in aberrant neoplastic tissue growth. In a variety of cancer types, cancer stem cells (cells within a cancer that have the ability to self-renew and drive tumorigenesis) have been proposed to modulate thyroid hormone’s effects through deiodinase and TR expression [16,17,18,19]. This interplay is thought to rely, in part, on Wnt signaling within cancer stem cells. Here, we review the effect of TH on cancer stem cell regulation and the current evidence for the role of Wnt pathway signaling in mediating tissue-specific, dynamic TH control.

### 2.2. Regulation of the Wnt Pathway by Thyroid Hormone Receptors

The actions of TH and Wnt signaling have been shown to reciprocally influence each other. In the current model of Wnt signaling, if Wnt ligand is absent, β-catenin is degraded through its association with a destruction complex that includes glycogen synthase kinase 3 (GSK3), casein kinase 1(CK1), adenomatous polyposis coli (APC), and Axin [20]. Wnt pathway activation is initiated upon binding of Wnt ligands to members of the Frizzled (FZD) and LRP5/6 co-receptor family. This process ultimately leads to receptor activation via a mechanism involving the cytoplasmic protein, Dishevelled (Dvl). As a consequence, β-catenin is stabilized and translocated to the nucleus, where it mediates a Wnt-specific transcriptional program. The current evidence indicates that the interaction between Wnt signaling and TH signaling is likely context-dependent, and they act to either antagonize or potentiate each other’s functions in order to maintain homeostasis within different target tissues [17,21,22,23,24].

TH has been demonstrated to act on the Wnt pathway in tissues both during development and in neoplasia. In a rat pituitary model that is often used to study thyroid hormone and its receptors, activated TH, T3, induces cellular proliferation in pituitary cells and Wnt pathway silencing [24]. Specifically, following T3 stimulation, there is a decrease in levels of β-catenin and an increase in levels of Axin. Axin is part of the destruction complex responsible for ubiquitination of β-catenin. The group also demonstrates, using a luciferase reporter, that T3 treatment results not only in decreased β-catenin, but also in decreased transcriptional activity of the β-catenin–T-cell Factor (TCF) complex. It was proposed that translational and posttranslational mechanisms may regulate this Wnt silencing. This finding is opposite to the effect of T3 on colon cancer cells, where T3 exposure reduced tumor proliferation [18]. The contrasting effect of T3 on the behavior in colon cancer cells versus other tissues is speculated to be due to differences in the expression of deiodinases and TRs that may modify the oncogenic effects of Wnt signaling [18,23].

**Figure 2:**
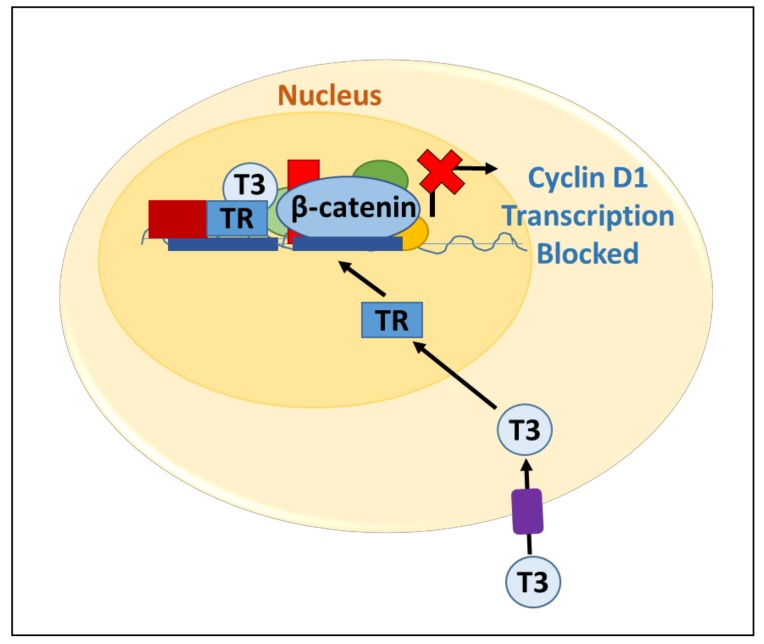
The effects of T3 on cyclin D1 transcription in colon cancer. T3 enters the target cell through membrane transport. T3 enters the nucleus and binds to the thyroid hormone receptor (TR). TR then recruits the co-activator complex. This complex then inhibits transactivation by β-catenin/TCF on the Cyclin D1 promoter.

Distinct mechanisms by which thyroid hormone receptors regulate Wnt signaling have been described. TRs can affect transcription at the level of Wnt target gene promoters. TRs heterodimerize and bind to TREs located in regulatory regions of target genes. In the T3-free state, TRs interact with transcriptional co-repressors to inhibit target genes. When T3 binds, however, co-repressors are displaced and co-activators are recruited [25]. Thus, transcription of TR target genes is monitored by a differential co-repressor–co-activator recruitment pathway. Natsume et al. have shown that in colon cancer, T3 binding to TRβ1 can suppress Wnt signaling by inhibiting β-catenin/TCF/LEF-1-mediated transcription of cyclin D1 (Figure 2) [26]. In addition to regulating the promoters of Wnt target genes, TRs can also bind directly to β-catenin. Guigon et al. used a mouse model of thyroid cancer to evaluate the interactions of TRβ and β-catenin [17]. They found that TRβ binds to β-catenin and that T3 binding disrupts this interaction, which enhances β-catenin proteasomal degradation and decreases Wnt signaling. Furthermore, using a TRβ^PV/PV^ mouse model in which the TRβ mutant is unable to bind T3, they demonstrated that β-catenin levels are elevated [17]. In summary, differential expression of TRs in tissues can lead to changes in Wnt/β-catenin signaling within target tissues via distinct mechanisms.

### 2.3. Control of Thyroid Hormone Function by the Wnt Pathway

The Wnt pathway has been shown to indirectly regulate TH function. Deiodinases are tightly controlled target genes of the Wnt pathway. Specifically, the management of D3 and D2 transcription is dynamically regulated by multiple signaling pathways, including the Wnt pathway [23]. The differential regulation of D2 and D3 is important during fetal development in protecting certain organs from T3 over-exposure; specifically, target organs can increase D3 expression to inactivate thyroid hormone and decrease D2 expression to prevent additional activation of thyroid hormone. One such T3-sensitive organ is the retina. During development of cones and color vision, D3 must be highly expressed in order to protect these cells from excessive T3 [21,22]. Elevated D3 also influences the development of the hypothalamic-pituitary-thyroid axis, as D3 knock-out mice have central hypothyroidism [27]. Studies of D3 knock-out in zebrafish have also revealed the significant contribution of D3 regulation to normal development [28].

The control of deiodinases by Wnt signaling is best described using a colon cancer model, where D3 expression is increased and D2 expression is decreased following Wnt stimulation [18]. D3 promoters are thought to be direct targets of Wnt signaling, where the β-catenin–TCF-4 complex binds to and regulates these promoters. D2 is also thought to be a direct target of Wnt signaling, but the molecular mechanisms of this regulation have yet to be elucidated. Indeed, transfection of a dominant-negative TCF-4 into a colorectal carcinoma cell line with constitutively active β-catenin led to increased D2 mRNA and decreased D3 mRNA [18]. Chromatin immunoprecipitation experiments have demonstrated that β-catenin associates with the promoter region of the D3 gene to mediate its transcription [18].

**Figure 3:**
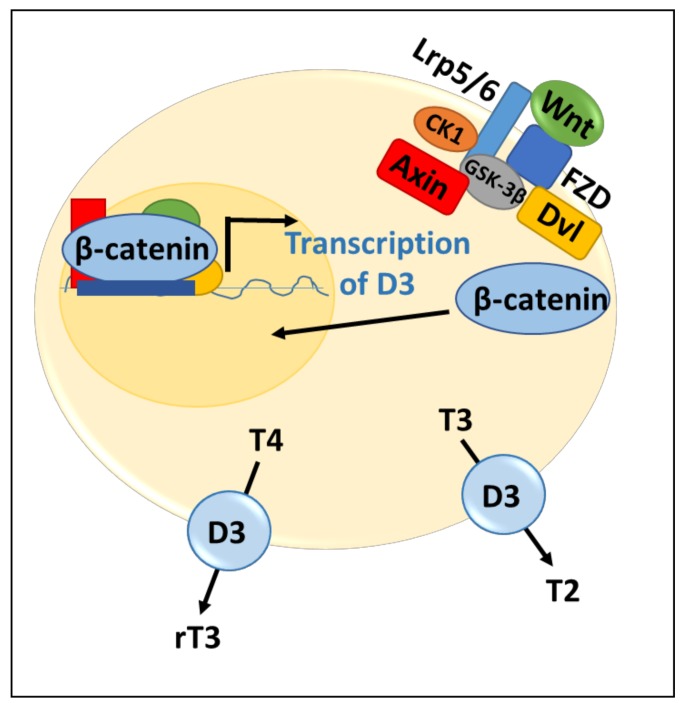
Wnt signaling pathway regulates deiodinases. Wnt ligand binds to the receptor, Frizzled (FZD), and the co-receptor, lipoprotein receptor-related protein 5 or 6 (Lpr5/6). Disheveled (Dvl) is then recruited, leading to phosphorylation of Lrp5/6 by casein kinase 1 (CK1) and glycogen-synthase kinase 3β (GSK-3β). Axin then binds, allowing for β-catenin to accumulate and translocate to the nucleus to activate transcription of D3. D3 levels then increase and lead to inactivation of T4 to reverse triiodothyronine (rT3) and T3 to T2.

Control of D3 expression by Wnt signaling in intestinal stem cells blocks differentiation and plays a role in colonic tumorigenesis. Intestinal stem cells are required to provide regeneration and maintenance of this epithelium [1,29]. This homeostatic process is regulated by the Wnt–β-catenin pathway, and over-activation of this pathway is a main driver of colon cancer [30]. Within the intestinal epithelium, β-catenin expression leads to D3 upregulation and D2 downregulation [18]. In the colonic epithelium, this control of D2 and D3 expression by the Wnt pathway has been shown to participate in colon cancer neogenesis (Figure 3) [18]. Specifically, induction of D3 by the Wnt pathway monitors the production of active TH to prevent differentiation of the neoplastic intestinal cells. Consistent with this, the addition of T3 to colon cancer cells reduces the proliferative capacity of the tumor [18] via its effects on differentiation.

## 3. Thyroid Gland Development and Adult Stem Cells

TH, which is secreted solely by the thyroid gland, is critical for homeostasis within an organism. The development and maintenance of the thyroid gland is essential for the upkeep of tissues throughout the body. Errors during thyroid development lead to congenital defects in the thyroid gland and the inability to form adequate thyroid hormone in children. Autoimmune diseases, iodine deficiency, and thyroid surgery result in insufficient thyroid hormone production in adults. While the presence of thyroid stem cells has recently been demonstrated [31,32], the pathways essential for stem cell function are still poorly understood. The Wnt signaling pathway is responsible for stem cell maintenance in many organs [5,6], and there is mounting evidence that it may also play a role in thyroid homeostasis via regulation of thyroid stem cells [7].

### 3.1. Thyroid Gland Development

The thyroid gland is composed of differentiated thyroid follicular epithelium that is organized in spherical follicles. This epithelium is made up predominantly of thyroid follicular cells responsible for the production of thyroid hormone [33]. These cells form spherical thyroid follicles that store and release thyroid hormone. Parafollicular or C cells are scattered in between these thyroid follicles and produce calcitonin. While these two cell types form a functional thyroid gland, their developmental origins are distinct.

Thyroid cells develop from two separate structures, the thyroid anlage and the ultimobrachial bodies. Thyroid follicular cells develop from the midline anlage (or diverticulum), which contains endodermal cells and is located in the embryonic mouth cavity. Cells from this foregut endoderm migrate caudally, bifurcate, and become the thyroid follicular cells. The ultimobrachial bodies are derived from the fourth pharyngeal pouch, which is responsible for C-cell development. The ultimobrachial bodies are transient structures located on either side of the embryonic neck. The fusion of the ultimobrachial bodies and thyroid lobes creates this dynamic organ with two distinct and intermixed populations of cells [34,35]. The thyroid gland also contains stromal fibroblasts, a network of capillaries, macrophages, and mast cells [35].

Interestingly, while TSH stimulation of the thyroid is critical for organ maintenance in the adult, fetal development of the thyroid gland is thought to occur without significant TSH stimulation [35,36,37]. The mechanisms that drive lobation of the thyroid are poorly understood. Similarly, the role of the interactions between endoderm and surrounding structures during organogenesis are still being discovered [38,39]. Functional differentiation of thyroid follicular cells does not begin until the cells have finished migration. The process of thyrocyte differentiation relies on signaling events mediated by bone morphogenetic protein (BMP44) and fibroblast growth factor (FGF2) in Xenopus, murine, and human cells [40]. Thyroid progenitor survival and development has been shown to be dependent on Nkx2.1a, Pax2a, Pax 8, Hhex, Titf1/Nkx2–1 (TTF-1), and Foxe1 (TTF-2) among others [41,42,43,44,45]. While the Wnt pathway is known to be activated in these progenitors based on upregulation of Wnt target mRNA, the role of Wnt signaling in thyroid development has not yet been determined [40].

### 3.2. Thyroid Gland Stem Cells

Dumont et al. originally proposed the existence of thyroid adult stem cells [46,47]. To date, the identification of thyroid adult stem cells remains challenging, as these cells are believed to be fairly rare in the thyroid. While thyroid cell turnover is quite low (estimated turnover rate of 5 times during a normal lifespan) [46], under certain conditions there is great capacity for thyroid tissue growth and regeneration, such as after partial thyroidectomy or following immune injury. More recently, Thomas et al. defined a population of cells in solid cell nests of normal thyroid that expresses markers of pluripotent cells, including Oct-4, Gata-4, Pax8, and HNF4α [31]. Chen et al. elucidated the robust regenerative capacity of the thyroid and the possibility of Oct-4^+^ adult thyroid stem cells in a murine autoimmune thyroiditis model of thyroid injury [32]. Finally, studies of side population cells (cells that extrude dyes, such as Hoechst 33342, using ATP binding cassette (ABC) transporter proteins) that express stem cell genes critical for self-renewal and pluripotency have been identified in mouse and human thyroid [48].

Recently, Kurmann et al. demonstrated the generation of mouse thyroid progenitor cells from mouse pluripotent stem cells. They showed that thyroid progenitors developed from embryonic stem cell-derived definitive endodermal cells following incubation with BMP4 and FGF2. More importantly, thyroid progenitor cells were generated from normal and disease-specific (hypothyroid) mouse- and human-induced pluripotent stem cells. This study is unique in its ability to generate T4 production from thyroid organoids, as T4 production had not been achieved in prior models. Injection of these organoids into hypothyroid mice resulted in restoration of T4 production [40]. However, further investigation is needed to decipher the number of stem cells required for thyroid tissue generation, their organization within the thyroid, and their normal function during growth and repair.

### 3.3. Wnt Signaling in Thyroid Stem Cells

There is evidence to suggest a role for Wnt signaling in thyroid homeostasis. Several Wnt pathway proteins are expressed in normal thyroid epithelium, including members of the Wnt gene family (*Wnt-2*, *Wnt-3*, *Wnt-4*, *Wnt-5a*, and *Wnt-10b*), the Frizzled gene family (*Fz-1*, *Fz-2*, and *Fz-6*), and the Dvl gene family (*Dvl-1*, *Dvl-2*, and *Dvl-3*) [49]. Using a cell-based luciferase reporter assay, Helmbrecht et al. demonstrated TCF/LEF transcriptional activation in thyroid cells [49]. The importance of Wnt signaling in the proliferation of human thyroid cells was also highlighted by Chen et al. In their study, GSK-3β gene silencing in primary human thyrocytes using adenoviral-interference resulted in increased expression of β-catenin and increased cellular proliferation [50].

Interestingly, Kurmann et al. performed microarray analysis of the mRNA expression profiles of thyroid progenitors and found activation of BMP, Wnt, epidermal growth factor, and FGF signaling pathways. BMP4 and FGF2 were sufficient to generate Nkx2-1^+^ thyroid progenitor cells with Tg, TSHR, NIS, and TPO expression, and the addition of Wnt3a to the cocktail did not alter the lineage specification [40]. The authors replicated this finding in both murine and Xenopus models and suggested that canonical Wnt signaling may not be necessary for this aspect of thyroid progenitor cell generation. Although Wnt signaling was not required for thyroid progenitor cell specification, their mRNA expression profiles were consistent with Wnt pathway activation; this upregulation implies a critical, but currently undefined, role of Wnt signaling in thyroid stem cell biology. In addition, this process of thyroid progenitor initiation is inefficient and requires GFP labeling and cell sorting. The longevity of these cells following transplantation is also not yet defined. Wnt signaling, while not necessary for thyroid specification, may play an essential function in stem cell survival and self-renewal. This possibility is evidenced by the fact that expression of Lgr5, a Wnt target gene that marks stem cells in a variety of tissues, is observed at both mRNA and protein levels in a normal thyroid gland [1,51].

Additional studies are needed to delineate the role of the Wnt pathway in thyroid progenitor function. Identification of Wnt ligands within the stroma of developing thyroid and adult thyroid could clarify the time points at which Wnt signaling is necessary for stem cell function. It will be important to evaluate which cells are responsible for secreting Wnt ligands as well as which cells are receiving the Wnt signal. Finally, identification of the location and organization of stem cells within the adult thyroid has been challenging. As *Lgr5* is a common stem cell marker in many tissues and a Wnt target gene, Lgr5 labeling could be useful in identifying stem cell niches within the adult thyroid gland.

## 4. Thyroid Cancer and the Role of Wnt Signaling

Approximately 100 million people in the United States have thyroid nodules, the majority of which are benign and 5–15% of which are malignant. Thyroid cancer is rapidly increasing in the United States with papillary thyroid carcinoma (PTC) as the most common subtype. While most patients are cured of their PTC following initial therapy, ~15% have disease recurrence, and ~10% have distant metastatic disease. Current diagnostic testing fails to predict a patient’s risk for metastatic or aggressive disease. Correct identification of patients who are at higher risk for recurrence and/or metastasis remains challenging and is an important goal for the field, as it would allow for a more appropriate selection of patients for surgical and radio-iodine therapy. An understanding of thyroid cancer stem cell biology would pave the way for tremendous advances in thyroid cancer diagnostics, prediction of metastatic and recurrent disease, and improved treatment algorithms to prevent unnecessary surgeries. Emerging data show that cells with stem-cell-like properties are present within numerous thyroid cancer cell lines and that Wnt signaling affects their function.

### 4.1. Evidence for Wnt Signaling in Thyroid Cancer Genesis

The strongest evidence for the role of Wnt signaling in thyroid cancer development is observed in familial thyroid cancer syndromes. Patients with Familial Adenomatous Polyposis (FAP), Gardner’s syndrome, and Turcot’s syndrome who carry a mutation in the APC gene have an increased risk of thyroid cancer, resulting in inappropriate Wnt pathway signaling [52,53]. These thyroid tumors, known as the cribiform-morular variant, have been shown to maintain heterozygosity of the APC mutation; however; they also carry stabilizing mutations of β-catenin as well as RET–PTC rearrangements [54,55,56]. RET–PTC fusions, frequently RET–PTC1 and RET–PTC3, are common in PTC. RET–PTC fusions lead to constitutive activation of the oncogene, RET, and stimulation of the MAPK and PI3K-AKT pathways [57,58]. Recent studies indicate that RET–PTC fusions also trigger the Wnt pathway by phosphorylating β-catenin at the Y654 tyrosine residue and inhibiting its turnover [59,60]. The genetic alterations identified in familial thyroid cancer syndromes strongly support a role for aberrant Wnt signaling in thyroid cancer development. However, the mutational landscape of thyroid cancer is complex, and the involvement of Wnt signaling in each thyroid cancer subtype will need to be clearly defined.

**Figure 4:**
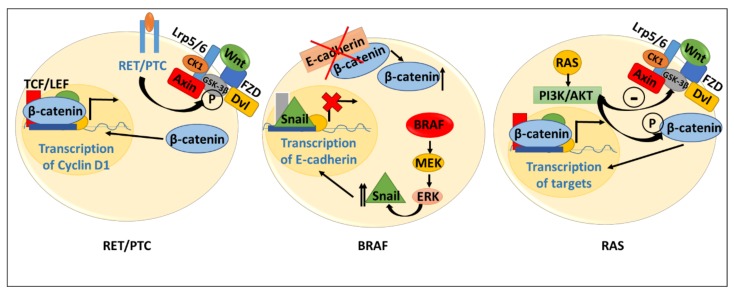
Proposed role of Wnt signaling in current papillary thyroid carcinomas (PTCs) with common driver mutations/alterations. In tumors with RET/PTC fusions, RET/PTC promotes the stabilization of β-catenin though phosphorylation of GSK-3β. This allows β-catenin to traffic to the nucleus and interact with TCF/LEF to promote cyclin D1 transcription. In tumors with BRAF^V600E^ mutation, BRAF^V600E^ is constitutively active and its signaling leads to an increased expression of Snail. Snail then acts to inhibit the transcription of E-cadherin by binding to the promoter and acting as a repressor. The decreased E-cadherin expression at the membrane leads to increased cytoplasmic β-catenin. Finally, in RAS-mutated tumors, RAS signaling through PI3K/AKT leads to stabilization of β-catenin through phosphorylation and through inhibition of GSK-3β. This allows β-catenin to traffic to the nucleus and promote transcription of target genes.

Well-differentiated thyroid carcinomas, such as PTC, represent some of the most common and least aggressive thyroid cancers. The metastatic activity of PTC has been shown to be dependent on E-cadherin downregulation and elevated levels of β-catenin [61]. PTCs are driven by three main genetic alterations: BRAF^V600E^ mutation, RET/PTC fusion, and RAS mutation (Table 2). Recent studies demonstrate that all three genetic subtypes ultimately stimulate the Wnt pathway via distinct mechanisms to promote tumorigenesis (Figure 4). As described above, RET–PTC fusion stimulates the Wnt pathway by promoting the stabilization of β-catenin [59,60,62].

BRAF-driven tumors have also been speculated to rely on Wnt signaling, although the details of this interaction remain to be determined. The BRAF^V600E^ mutation has been shown to activate the MAPK/ERK pathway, and there is evidence of cross-talk between MAPK/ERK signaling and the Wnt pathway in several tumor types, including lung cancer and melanoma [63,64,65]. In thyroid tumors, elucidation of the interaction between BRAF and Wnt signaling is just beginning to emerge, and BRAF mutations in PTC appear to cause downregulation of E-cadherin, potentially promoting Wnt signaling via a mechanism involving increased levels of cytoplasmic β-catenin [66]. Perhaps more convincing is the study by Park et al. of gene expression in The Cancer Genome Atlas, in which they observed that PTCs with extrathyroidal tumor extension exhibited aberrant Wnt pathway expression as evidenced by upregulation of SFRP2, SFRP4, Wnt-7A, and Wnt-2 and downregulation of Wnt-4 [67]. As BRAF mutations are associated with more frequent extrathyroidal extension, a correlation between Wnt-4 and mutational phenotype was evaluated. They found that Wnt-4 was reduced in BRAF mutant tumors, but unchanged in RAS mutant samples [67].

RAS-driven thyroid tumors have been proposed to regulate canonical (Wnt/β-catenin) and non-canonical Wnt signaling via multiple mechanisms. In contrast to the study by Park et al. that found Wnt-4 expression to be unchanged in RAS-mutant tumors, De Menna et al. demonstrated Wnt-4 downregulation by RAS [68]. This group showed enhanced rat thyrocyte motility following expression of oncogenic RAS that strongly correlated with Wnt-4 downregulation. Conversely, expression of Wnt-4 inhibited RAS-dependent motility. The authors suggested that RAS-mediated suppression of Wnt-4 was due to induction of miR-24 by RAS [68].

In thyroid tumors driven by RAS mutation, activation of PI3K/AKT caused a reduction in E-cadherin levels, increased nuclear β-catenin localization, and activation of the Wnt pathway. Nuclear localization of β-catenin was proposed to be mediated by its phosphorylation by AKT at Ser552. The role of Wnt signaling in thyroid cancer cell growth was confirmed by the demonstration that short hairpin RNA (shRNA)-mediated silencing of β-catenin resulted in decreased proliferation and promotion of cellular senescence as measured by an increase in β-galactosidase activity [69]. Finally, Cho et al. showed that the soluble inhibitor, Dickkopf-1, could block survival and migration of PTC cells via inhibition of Wnt signaling and E-cadherin upregulation [70].

Follicular thyroid carcinoma (FTC), another well-differentiated carcinoma, has also been found to be regulated by Wnt signaling. Using the mutant TRβ^PV/PV^ mouse model of FTC, it was demonstrated that Wnt signaling is elevated in FTC via a mechanism involving stabilization of β-catenin as described earlier. In addition, the TRβ^PV/PV^ mutant was also shown to interact with PI3K to activate AKT signaling [71] to promote the phosphorylation of β-catenin at Ser522, thereby enhancing its cytoplasmic and nuclear accumulation [72]. TRβ^PV/PV^ mice also have increased TH levels, which has been suggested to further activate AKT in these tumors. Elevated T3 is proposed to bind to the TH membrane receptor, integrin αvβ3, so as to promote extranuclear signaling [73]. Lu et al. hypothesized that integrin αvβ3 signaling leads to decreased PTEN activity, increased PIP3, increased phosphorylated AKT, and ultimately enhanced Ser522 β-catenin phosphorylation [72].

Anaplastic thyroid carcinomas (ATC) are some of the most aggressive and lethal cancers. These cancers exhibit p53 pathway deregulation and stabilizing β-catenin (CTNNB1) mutations, which contribute to their aggressive behavior (Table 2) [74]. In one investigation involving 22 patients with ATC, ~40% of the tumors were reported to have nuclear β-catenin staining. Furthermore, 4.1% of tumors had stabilizing β-catenin mutations, 9.0% had APC mutations, and ~82% had Axin mutations [75]. Several groups have shown that β-catenin and E-cadherin functions are essential for ATC survival and migration. Indeed, loss of E-cadherin expression has been found to be a poor prognostic marker in thyroid cancer [76,77]. A study of ATC cell lines demonstrated that addition of the tyrosine kinase inhibitor, imatinib mesylate, led to decreased β-catenin, increased β-catenin/E-cadherin complex formation, and diminished aggressive tumor behavior [78]. The classical non-canonical Wnt ligands, Wnt-5a and Wnt-4 [79], have been shown to play a role in anaplastic thyroid carcinoma malignancy. Wnt-5a normally is expressed in follicular adenomas and well-differentiated thyroid carcinomas, such as PTC and FTC, and has been proposed to act as a tumor suppressor [80]. Loss of Wnt-5a expression, as is often seen in ATC, is associated with a more aggressive, malignant phenotype [80]. In addition, Wnt-4 is downregulated in ATC [68]. In summary, there is compelling evidence that thyroid carcinomas are dependent on Wnt signaling for growth and proliferation, with elevated levels of signaling associated with poorly differentiated disease.

### 4.2. Evidence for Thyroid Cancer Stem Cells

Thyroid “cancer stem cells” have been an important area of research and a topic of debate. Indeed, these cancer cells with stem-cell-like properties are thought to be responsible for tumor growth and, due to their resistance to chemotherapeutic agents, are also believed to be responsible for tumor recurrence. The existence of such cancer cells with a progenitor phenotype in thyroid tumors has not yet been proven, but data are beginning to emerge indicating that they may indeed be crucial for thyroid cancer pathogenesis. There are multiple hypotheses regarding the origin of cancer stem cells in the thyroid. One is that well-differentiated thyroid cancer cells transform into more de-differentiated cell types through a series of mutations and possibly epithelial-to-mesenchymal transition [34,85]. Another proposes that thyroid cancer arises from residual fetal cells with stem-cell marker expression that reside within solid cell nests in the thyroid [34,86,87,88]. The third model suggests that cancer stem cells could arise from resident thyroid stem cells that accumulate a series of genetic alterations [89,90]. This theory has gained considerable attention in recent years and is particularly attractive as it combines the concept of cancer stem cells with the model of multiple mutational events leading to cancer. This would also explain why studies of more aggressive thyroid cancer subtypes have more genetic alterations and more malignant behavior.

Much attention has focused on the search for stem cells within previously characterized thyroid cancer cell lines. Side population cells (described above) that exhibit stem-cell characteristics and can give rise to both side population and non-side population cells have been described. For example, one study identified an Oct-4+ side population of cells within an anaplastic thyroid carcinoma that were multi-drug resistant due to the ABCG2 multi-drug resistance transporter [91]. Evaluation of the gene expression of these side population cells by quantitative real-time RT-PCR revealed increased expression of FZD5, a receptor for Wnt-5a in the non-canonical Wnt pathway, as compared to non-side population cells [48].

### 4.3. Wnt Signaling in Thyroid Cancer Stem Cells

While studies of driver mutations in thyroid cancer have implicated Wnt signaling in pathogenesis, estrogen stimulation may also activate Wnt signaling. Although both women and men rely on TH for homeostasis, women have a substantially higher burden of thyroid disease as well as a higher incidence of thyroid cancer. One reason for this disparity involves the difference in hormone expression and regulation in males versus females. Circulating estrogens are thought to increase thyroid disease in women through their activation of the PI3K pathway and repression of p27 expression [92]. Reports suggest that estrogen stimulation also regulates thyroid cancer stem cell function. Xu et al. demonstrated that stem cells from thyroid goiters had enhanced organoid formation with stimulation by 17β-estradiol (E2) [93]. One potential mechanism may be via the Wnt pathway, as Koumenko et al. have described the interaction of β-catenin with an estrogen receptor, ER-α, in connecting estrogen stimulation to Wnt activation [94]. Specifically, this group found that ER-α and β-catenin existed in the same immunocomplexes and were reciprocally recruited to the promoters of target genes. Consistent with these findings, other studies have suggested that E2 stimulation is associated with increased β-catenin levels as well as increased invasion and migration of thyroid tumors [95]. A model of estrogen-stimulated Wnt pathway activation in thyroid stem cells is particularly attractive, as Wnt signaling is a critical regulator of cancer pathogenesis in other organs, and its stimulation by estrogen could help to explain the gender disparity of thyroid disease.

Thyroid cancer stem cells may arise from differentiated thyroid cancer cells that undergo epithelial-to-mesenchymal transition (EMT) to a de-differentiated state via a mechanism involving the Wnt pathway. Lan et al. proposed that EMT could be induced through HIF-1α and that activation of the Wnt signaling pathway accompanied this transition [85]. While this model may not represent the true origin of thyroid cancer stem cells, it highlights the importance of Wnt signaling in the maintenance of thyroid cancer progenitor cells. Todaro et al. reported the most convincing evidence for the role of Wnt signaling in thyroid cancer stem cells. They identified progenitor cells with stem-cell-like properties within thyroid cancer cell lines and found that these cells accumulated nuclear β-catenin and possessed a migratory capability. Indeed, cells with increased migration showed nuclear β-catenin and a loss of E-cadherin [16].

## 5. Conclusions

A considerable body of work published in recent years has enhanced our understanding of thyroid stem cell biology. There is now convincing evidence for the existence of adult stem cells in the thyroid gland. These cells are likely responsible for thyroid regeneration following hemi-thyroidectomy or injury from autoimmune attack and emerging data suggest that Wnt signaling is likely required for their maintenance. Further investigation is needed to delineate the precise location of these stem cells within thyroid tissue, the timing and function of Wnt signaling within these stem cells, and the source of the Wnt ligands.

Recent studies on thyroid cancer stem cells have also provided evidence for their potential roles in metastasis and recurrence. Indeed, while most thyroid cancer is cured following standard therapy, a subset of patients has metastatic and/or recurrent aggressive disease that is driven, at least to some extent, by Wnt signaling, although the exact role of this pathway in each thyroid cancer subtype remains unclear. Stem-cell markers, such as Lgr5 (also a Wnt target gene), may be useful for locating thyroid cancer stem cells in order to verify their presence and disclose their organization within thyroid tumors.

Modulation of Wnt signaling within both normal and neoplastic thyroid tissue may help to further define the role of this signaling pathway in thyroid homeostasis and malignancy. Targeting Wnt in patients with thyroid disease could have tremendous implications for therapy. Small molecule Wnt activators, such as lithium (a GSK-3β inhibitor), could increase stem cell function and lead to thyroid tissue regeneration following surgery or autoimmune injury. Lithium chloride (LiCl) is known to cause thyroid tissue growth and goiter formation [96,97,98]. This effect is due, at least in part, to stimulation of the Wnt pathway [98]. However, increased thyroid tissue is not desirable if it is not producing thyroid hormone. As lithium is also known to inhibit the synthesis, release, and deiodination of thyroid hormone, its administration and stimulation of thyroid growth may not lead to decreased hypothyroidism [96,99]. In addition, Wnt blockade, rather than enhancement, may be critical in Hashimoto’s disease, as these patients have a higher risk of developing thyroid cancer. In fact, the inhibition of Wnt signaling in patients with longstanding Hashimoto’s thyroiditis may be an important strategy for the prevention of thyroid malignancy. Additional research is needed to understand the complex role of Wnt signaling in thyroid stem cell biology and thyroid homeostasis and improve the treatment of thyroid disease.

Given the accumulating evidence for Wnt signaling in thyroid cancer (particularly the aggressive and malignant forms), a thorough evaluation of the roles of Wnt signaling in thyroid tumor progression is needed. Demonstration that Wnt signaling plays a role in thyroid cancer can be readily performed by simple knockdown/knockout studies with RNA interference (RNAi) or CRISPR/Cas9 editing technologies. Alternatively, various small molecule inhibitors have been developed that are in various stages of clinical trials, including Wnt ligand inhibitors (currently in Phase I Clinical Trials) [100] and antagonists of the TCF/β-catenin transcriptional complex (currently in Phase I/II Clinical Trials) [101]. These inhibitors could be tested for their capacity to inhibit thyroid cancer growth and metastasis in cell-based and animal models. Perhaps the most promising use of Wnt inhibitors may be for patients with extremely treatment-resistant anaplastic thyroid carcinomas which carry activating Wnt pathway gene mutations. Anaplastic thyroid carcinomas patients have an abysmal survival rate (4 months), and anti-Wnt therapy may be particularly beneficial to this subset of thyroid cancer patients. In conclusion, Wnt blockade in thyroid cancer stem cells has great potential to provide critical information on tumorigenesis and to inform strategies for targeting these cells in thyroid cancer treatment.

## Figures and Tables

**Table 1:** Deiodinase expression in human tissue and its role in normal tissue function as well as in hyper- and hypothyroid states.

Tissue	Deiodinase Expression	Normal Function	Hyperthyroidism	Hypothyroidism
**Thyroid Liver Kidney Skeletal muscle**	D1	Production of T3 into plasma	Increased function	Decreased function
**Brain**	D2	Production of T3 into plasma and local tissues	Decreased function	Increased function
**Placenta Brain**	D3	Degradation of T3	Increased function	Decreased Function

T3: thyroxine.

**Table 2:** Non-medullary thyroid cancer sub-types and their most common mutations/signaling pathways. Frequency: >40% = +++; 10–40% = ++; <10% = +.

Thyroid Cancer	Mutation/Alteration	Frequency	Signaling Pathways	References
**Papillary Thyroid Carcinoma**	BRAF	+++	MAPK	Nikiforov 2008 [81] Ishigaki 2002 [82] Garcia-Rostan 2001 [74] Rezk 2004 [83]
	RET/PTC	++	PI3K/AKT	
	RAS	++	MAPK	
			PI3K/AKT	
	TRK	+	MAPK	
			PI3K/AKT	
	CTNNB1 dysregulation	++	Wnt Signaling	
**Follicular Thyroid Carcinoma**	RAS	+++	MAPK	Nikiforov 2008 [81] Farrow 2003 [84] Garcia-Rostan 2001 [74] Rezk 2004 [83]
			PI3K/AKT	
	PAX8-PPARγ	++	PI3K/AKT	
	PIK3CA	+	PI3K/AKT	
	PTEN	+	PI3K/AKT	
	CTNNB1 dysregulation	++	Wnt Signaling	
**Poorly Differentiated Thyroid Carcinoma**	RAS	++	MAPK	Nikiforov 2008 [81] Garcia-Rostan 2001 [74]
			PI3K/AKT	
	CTNNB1	++	Wnt signaling	
	TP53	++	p53 signaling	
	BRAF	++	MAPK	
**Anaplastic Thyroid Carcinoma**	TP53	+++	p53 signaling	Nikiforov 2008 [81] Kurihara 2004 [75] Garcia-Rostan 2001 [74]
	CTNNB1	+++	Wnt signaling	
	AXIN	+++	Wnt signaling	
	RAS	+++	MAPK	
			PI3K/AKT	
	BRAF	++	MAPK	
